# Electronic Health Records for Population Health Management: Comparison of Electronic Health Record–Derived Hypertension Prevalence Measures Against Established Survey Data

**DOI:** 10.2196/48300

**Published:** 2024-03-13

**Authors:** Katie S Allen, Nimish Valvi, P Joseph Gibson, Timothy McFarlane, Brian E Dixon

**Affiliations:** 1 Regenstrief Institute, Inc Indianapolis, IN United States; 2 Richard M Fairbanks School of Public Health Indiana University–Purdue University Indianapolis Indianapolis, IN United States

**Keywords:** public health informatics, surveillance, chronic conditions, electronic health record, health management, hypertension, surveillance, public health, prevalence, population-based survey

## Abstract

**Background:**

Hypertension is the most prevalent risk factor for mortality globally. Uncontrolled hypertension is associated with excess morbidity and mortality, and nearly one-half of individuals with hypertension do not have the condition under control. Data from electronic health record (EHR) systems may be useful for community hypertension surveillance, filling a gap in local public health departments’ community health assessments and supporting the public health data modernization initiatives currently underway. To identify patients with hypertension, computable phenotypes are required. These phenotypes leverage available data elements—such as vitals measurements and medications—to identify patients diagnosed with hypertension. However, there are multiple methodologies for creating a phenotype, and the identification of which method most accurately reflects real-world prevalence rates is needed to support data modernization initiatives.

**Objective:**

This study sought to assess the comparability of 6 different EHR-based hypertension prevalence estimates with estimates from a national survey. Each of the prevalence estimates was created using a different computable phenotype. The overarching goal is to identify which phenotypes most closely align with nationally accepted estimations.

**Methods:**

Using the 6 different EHR-based computable phenotypes, we calculated hypertension prevalence estimates for Marion County, Indiana, for the period from 2014 to 2015. We extracted hypertension rates from the Behavioral Risk Factor Surveillance System (BRFSS) for the same period. We used the two 1-sided *t* test (TOST) to test equivalence between BRFSS- and EHR-based prevalence estimates. The TOST was performed at the overall level as well as stratified by age, gender, and race.

**Results:**

Using both 80% and 90% CIs, the TOST analysis resulted in 2 computable phenotypes demonstrating rough equivalence to BRFSS estimates. Variation in performance was noted across phenotypes as well as demographics. TOST with 80% CIs demonstrated that the phenotypes had less variance compared to BRFSS estimates within subpopulations, particularly those related to racial categories. Overall, less variance occurred on phenotypes that included vitals measurements.

**Conclusions:**

This study demonstrates that certain EHR-derived prevalence estimates may serve as rough substitutes for population-based survey estimates. These outcomes demonstrate the importance of critically assessing which data elements to include in EHR-based computer phenotypes. Using comprehensive data sources, containing complete clinical data as well as data representative of the population, are crucial to producing robust estimates of chronic disease. As public health departments look toward data modernization activities, the EHR may serve to assist in more timely, locally representative estimates for chronic disease prevalence.

## Introduction

Hypertension is the most prevalent risk factor for mortality throughout the world [1]. The condition is characterized by elevated systolic blood pressure (>140 mm Hg) or diastolic blood pressure (>90 mm Hg) [2]. An estimated 1 out of 3 adults in the United States has been diagnosed with hypertension, which translates to almost 75 million Americans [2]. This results in substantial use of health care services and medications, as well as lost wages [3,4]. The estimated direct and indirect costs of hypertension exceed US $48 billion each year in the United States [5]. In concurrence with economic loss, uncontrolled hypertension is associated with excess morbidity and mortality, and nearly one-half of individuals with hypertension do not have the condition under control [2].

Uncontrolled hypertension is associated with an increased risk of coronary heart disease, stroke, and kidney disease, which are the 3 leading causes of death in the United States [5]. Hypertension is a comorbid condition for nearly 70% of individuals who have their first myocardial infarction and almost 80% of those who have their first stroke [6]. Additionally, hypertension is associated with an excess risk of severe COVID-19 illness with a risk of hospitalization more than double that of nonhypertensive individuals [7]. The association with increased morbidity and mortality is a critical public health concern given the high prevalence of the condition. To address this increasing public health concern, public health programs and policies aimed at reducing morbidity, mortality, and costs associated with hypertension are required. To create these policies, public health departments are reliant on timely, accurate, stable estimates of disease prevalence. This is required both for timely detection and effective evaluation.

Identifying the prevalence of hypertension as well as measuring hypertension control at the community level remains a challenge for local health departments. While clinical guidelines from the National Quality Forum and others (eg, Centers for Disease Control and Prevention and Healthcare Effectiveness Data and Information Set) exist [8], measurement happens at the level of a provider or health system as opposed to the community. Public health departments typically rely on surveys for measuring community-level estimates of hypertension. However, surveys have known limitations including cost and timeliness due to long gaps between data collection and when results are available. Additionally, the local samples are insufficiently small for precise estimates within communities and subpopulations (ie, wide CIs). Therefore, local health departments seek alternative methods for obtaining timely, complete, accurate, and precise information about the prevalence of chronic conditions such as hypertension and measures of control for individuals with chronic illness.

Since the passing of the Health Information Technology for Economic and Clinical Health Act of 2009, electronic health record (EHR) systems have become more common, representing a potential data source for chronic disease surveillance. As of 2016, over 70% of ambulatory providers use EHR systems [9]. As health care organizations increasingly capture data from routine health care visits in EHR systems, national initiatives, including the digital Learning Health System of the National Academy of Medicine [10] and the Robert Wood Johnson Foundation’s data for health [11], aim to leverage such data to improve the delivery of health care and community health outcomes. The hope is that by leveraging existing digital data sources, public health agencies may access more timely and precise information to assess and improve health in their communities.

While there exists much optimism about EHR systems’ ability to provide timely, complete, and accurate estimates for hypertension and other chronic diseases, evidence to date has been mixed. In a systematic review of the quality of data used for quality-of-care measurement, the completeness of data varied “substantially across studies,” ranging from 0.1% to 51% for blood pressure and from 10% to 38% for smoking status [12]. Missing data ranged between 24% and 38% for cholesterol; 3% and 31% for blood pressure; and 5% and 23% for blood glucose (hemoglobin A_1C_) [12].

Despite these challenges, EHR data may be useful for community health surveillance. More recent work by the New York City (NYC) Department of Mental Health and Hygiene shows promising results in using EHR data for measuring the prevalence and control of chronic diseases [13,14]. By querying EHR systems in primary care practices representing 15% of the city’s population, the health department found prevalence rates were in line with community-based surveys for diabetes, obesity, hypertension, and smoking even when the survey respondents were limited to those who had received primary care in the prior year (NYC Health and Nutrition Examination Survey and the NYC Community Health Survey [15]). More recent studies give hope that EHR data could be used by health departments to improve the timeliness and precision of their community health assessments [16-18].

Given limited prior evidence, we sought to validate computable phenotypes for hypertension using EHR data available through a community-based health information exchange (HIE) network. The use of HIE data was selected to examine data representing a geographic community rather than the population of a single health system. Our goal is to identify methods that can be leveraged by health departments for the surveillance of chronic illnesses and the calculation of control measures.

Accordingly, the objective of this analysis was to analyze the equivalence of EHR-based methods for deriving the prevalence of hypertension compared to an established community survey. To facilitate this analysis, 6 distinct EHR-based phenotypes for hypertension were used to establish prevalence rates in 1 county. These rates were then tested for equivalency with the prevalence calculated by a national survey. We hypothesized that at least 1 of the selected phenotypes would produce equivalent estimates.

## Methods

### Data Sources

#### Indiana Network for Patient Care

The primary data source was the Indiana Network for Patient Care (INPC), a regional HIE with data covering emergency department visits, hospital admissions, and large outpatient health care clinics from across the state. Data were supplemented with direct extracts from 1 health system to provide additional vital measurements and medication data that were not currently shared with the INPC. For this study, the focus was Marion County, Indiana, which is the county containing the largest city, Indianapolis, and we leveraged 3 of the 5 major health systems. Using the 3 health systems ensures that approximately 780,000 (80%) of the population of Marion County was captured for this study. According to the 2010 census, Marion County had a resident population of 977,203 with a racial composition of 30% Black or African American, 11.6% Hispanic, and 61.9% White.

Data were extracted for all adults (at least aged 18 years as of January 1, 2014) living in Marion County who sought care (outpatient, inpatient, or emergency department encounters) at 1 of the 3 large integrated delivery networks that connect to the INPC between January 1, 2014, and December 31, 2015. We used 2 years of data to capture a representative number of clinical encounters since individual health care use may not occur annually. This period was used due to the availability of comprehensive data from 3 of the 5 major health systems in the area. Given the period covered in this data set, the data do not establish current prevalence rates for Marion County but rather serve as an example for the surveillance methodology deployed. The algorithms to detect hypertension in the community were implemented on the data set, which contained diagnosis codes, vital measurements, and medications.

#### Behavioral Risk Factor Surveillance System

For the gold standard comparison, we used the Behavioral Risk Factor Surveillance System (BRFSS)—the US national survey related to health-related behaviors, chronic health conditions, and the use of preventive services. The prevalence estimates produced by the BRFSS are carefully developed, validated, and weighted to minimize biases in response or coverage [19]. The BRFSS collects data in all 50 states, the District of Columbia, and territories. However, for small geographics (eg, county) or population subgroups, the BRFSS is imprecise with large CIs. For this study, the data related to the 2015 prevalence of hypertension in Marion County, Indiana, was used.

### Measures

To facilitate analysis, BRFSS prevalence measures were compared to EHR-based measures extracted from the HIE. The 2015 BRFSS results include an overall hypertension prevalence rate as well as rates by age, race, and gender for Marion County. These measures were extracted from the US Centers for Disease Control and Prevention website [20].

The computable phenotypes used for this study were previously developed and reported separately [21]. Briefly, 6 phenotypes for hypertension were developed using algorithms (or rules) executed using 1 or more types of structured EHR data. These rules were validated using chart review to calculate sensitivity, specificity, and positive predictive value [21]. Defining multiple permutations allowed for evaluating the best-performing phenotype. The phenotypes are as follows:

- P1: clinical diagnostic codes only (in which an individual has either 1 inpatient or 1 outpatient encounter documenting a hypertension diagnosis)
- P2: vital statistics only (in which an individual has at least 1 blood pressure reading above the hypertension threshold)
- P3: vital statistics only (in which an individual has at least 2 blood pressure readings above the hypertension threshold)
- P4: clinical diagnosis and vital statistics (P1 and P2)
- P5: clinical diagnosis and vital statistics (P1 and P3)
- P6: Inclusive of P1-P5 and medications (P1, P2, or the use of hypertension medication)

Using the 6 different EHR-based computable phenotypes, we calculated hypertension prevalence estimates from data for residents of Marion County, Indiana, from the years 2014 and 2015. Prevalence was calculated as the number of persons with data satisfying the given phenotype divided by the number of persons with any HIE record for a health care encounter.

### Ethical Considerations

Exempt approval for this study was received by the Indiana University Institutional Review Board (1701925087).

### Statistical Analysis

Demographics for the INPC-derived cohort were calculated using P6, which is the broadest and most sensitive phenotype [21]. Using the estimates for Marion County outlined above, equivalency testing was performed. Equivalence testing examines whether 2 independent statistics are similar enough to be treated as though they are equivalent. The null hypothesis is that the statistics differ by at least a specified amount. If the test results in a P value <.05, then the null hypothesis is rejected with a conclusion that the 2 statistics differ by less than the specified amount. We used the two 1-sided *t* test (TOST) to test equivalence between BRFSS- and INPC-based prevalence estimates. The TOST was performed at the overall level as well as stratified by age, gender, and race. The TOST was performed with 80% and 90% CI. As with other large national surveys, BRFSS estimates have wide CIs. Accordingly, widening the TOST analysis threshold was considered to account for the wide CIs within the BRFSS data set compared to the small CIs associated with the larger INPC data set. The 95% CI of the BRFSS overall hypertension estimates for Marion County is 7-7.5 percentage points wide. The stratified BRFSS hypertension rates are slightly wider. Accordingly, our specified amounts align with the CIs for the BRFSS. This study used SAS (version 9.4; SAS Institute Inc) and Excel 365 (Microsoft) for analyses.

## Results

The demographics for the BRFSS and INPC cohorts are presented in Table 1. The EHR-based phenotypes were calculated from INPC data for 548,232 patients, which was the number of adult patients with at least 1 clinical encounter during the period. Overall, the cohort was 61.2% (n=335,548) women and 27% (n=148,117) Black or African American. Of the total INPC-derived cohort, 210,764 (38.4%) patients were identified as hypertensive by phenotype P6, which is the broadest—and most sensitive—definition of hypertension according to Valvi et al [21]. The INPC-derived hypertension cohort was 57.6% (121,307/210,764) women and 33.2% (70,060/210,764) Black or African American. The BRFSS-derived hypertensive cohort was 55.2% (197/357) women and 17.6% (63/357) Black or African American. The INPC cohort was more racially diverse than the BRFSS cohort overall. The BRFSS cohort had less representation of the younger population and overrepresentation of those aged 65 years and older.

**Table 1:** Cohort demographics^a^.

Demographics			Overall population				Hypertensive population		
			BRFSS^b^ (n=934), n (%)		INPC^c^ (n=548,232), n (%)		BRFSS (n=357), n (%)		INPC (n=210,764), n (%)
**Gender**									
	Women	524 (56.1)		335,548 (61.2)		197 (55.2)		121,307 (57.6)	
	Men	410 (43.9)		212,684 (38.8)		160 (44.8)		89,457 (42.4)	
**Race**									
	Black	152 (16.7)		148,117 (27)		63 (17.6)		70,060 (33.2)	
	White	702 (75.2)		308,213 (56.2)		273 (76.6)		120,832 (57.3)	
	Other	80 (8.6)		91,902 (16.8)		21 (5.9)		19,872 (9.4)	
**Age group (y)**									
	18-39	197 (21.1)		214,655 (39.2)		24 (6.7)		52,777 (25)	
	40-64	406 (43.5)		240,064 (43.8)		136 (38.1)		101,416 (48.1)	
	65+	331 (35.4)		93,513 (17)		197 (55.2)		56,571 (26.8)	

^a^Table 1 contains gender, race, and age counts and percentages for each of the cohorts. The cohorts include the overall population for both BRFSS and INPC as well as the hypertensive population.

^b^BRFSS: Behavioral Risk Factor Surveillance System.

^c^INPC: Indiana Network for Patient Care.

The TOST analysis was undertaken at both the 90% and 80% CIs. The TOST analysis at the 90% CI resulted in 2 phenotypes (P2 and P5) having statistically significant results, indicating their equivalency to BRFSS estimates, or, more specifically, given the assumptions of this analysis, it is at least 90% likely that hypertension prevalence estimates from the BRFSS and phenotypes P2 and P5 will differ by no more than 5 percentage points. However, performance in the stratified groups was much poorer with statistical significance for women only in phenotypes P1 and P4. By the nature of TOST, the wider an estimate’s CI, the less chance that the null hypothesis will be rejected; some stratified groups have CIs so wide that their TOSTs had zero power. The analysis at the 80% CI yielded statistically significant results across multiple phenotypes. At the 80% CI, phenotypes P2, P3, and P5 showed equivalency overall, with P2 and P5 also showing equivalence in 9 of the demographic subsets and P3 showing equivalence in 7 of those subsets. Tables 2-4 depict the full 80% CI analysis for P2, P3, and P5. All remaining analyses are included in the Multimedia Appendices 1 and 2.

**Table 2:** Full 80% CI analysis for phenotype 2, with overall ≥1 vitals indicated. This table depicts all analytical results for P2 at the 80% CI.

Characteristic			BRFSS^a,b^, n/N (%)		INPC^c,d^, n/N (%)		%Δ^e^ (Δ80% CI)
Overall			235/934 (28.4)		159,330/548,298 (29.1)		0.7 (–1.8 to 3.1)^f^
**Gender**							
	Men	127/410 (31)		66,758/212,684 (31.4)		0.4 (–10.6 to 11.4)	
	Women	137/524 (26.1)		92,570/335,548 (27.6)		1.5 (–6.6 to 9.6)^f^	
**Race**							
	Black or African American	54/152 (35.7)		57,026/148,120 (38.5)		2.8 (–3.3 to 8.9)^f^	
	White	187/702 (26.6)		89,205/308,224 (28.9)		2.3 (–0.3 to 5)^f^	
	Other	18/80 (22.6)		13,099/91,954 (14.2)		–8.4 (–15 to –1.7)	
**Age group (y)**							
	18-39	21/197 (10.8)		49,634/214,685 (23.1)		12.3 (9.2 to 15.4)	
	40-64	133/406 (32.8)		76,795/240,084 (32)		–0.8 (–4.5 to 2.9)^f^	
	65+	204/331 (61.6)		31,238/88,569 (35.3)		–26.3 (–30 to –22.6)	
**Men** **by race**							
	Black or African American	24/60 (40.6)		22,226/56,004 (39.7)		–0.9 (–7.1 to 5.2)^f^	
	White	91/314 (29.1)		38,832/120,672 (32.2)		3.1 (–1 to 7.2)^f^	
	Other	9/36 (24.1)		5,700/36,008 (15.8)		–8.3 (–18.1 to 1.6)	
**Women** **by race**							
	Black or African American	30/92 (32.2)		34,800/92,113 (37.8)		2.5 (–0.9 to 5.8)^f^	
	White	95/388 (24.4)		50,373/187,541 (26.9)		–7.6 (–16.3 to 1.1)	
	Other	9/44 (20.8)		7,379/55,894 (13.2)		5.6 (–2.4 to 13.5)	
**Men** **by age group (y)**							
	18-39	18/99 (18.5)		20,478/77,992 (26.3)		7.8 (2.3 to 13.3)	
	40-64	56/178 (31.2)		33,928/98,778 (34.3)		3.1 (–2.2 to 8.5)^f^	
	65+	90/133 (67.4)		11,957/34,606 (34.6)		–32.8 (–38.6 to –27.1)	
**Women** **by age group (y)**							
	18-39	3/98 (3.4)		29,155/136,663 (21.3)		11.5 (15.6 to 20.2)	
	40-64	78/228 (34.2)		42,866/141,286 (30.3)		–3.9 (–9.1 to 1.4)^f^	
	65+	114/198 (57.5)		19,281/53,954 (35.7)		–21.8 (–29.1 to –14.5)	

^a^BRFSS: Behavioral Risk Factor Surveillance System.

^b^Sample size=934.

^c^Indiana Network for Patient Care.

^d^Sample size=548,298.

^e^Δ: mean difference.

^f^Behavioral Risk Factor Surveillance System and Indiana Network for Patient Care phenotypes were determined as statistically equivalent by the two 1-sided *t* test method.

**Table 3:** Phenotype 3, overall ≥2 vitals indicated. This table depicts the full analytical results for P3 at the 80% CI.

Characteristic			BRFSS^a,b^, n/N (%)		INPC^c,d^, n/N (%)		%Δ^e^ (Δ80% CI)
Overall			235/934 (28.4)		122,051/548,298 (22.3)		–6.1 (–8.6 to –3.7)^f^
**Gender**							
	Men	127/410 (31)		50,997/212,684 (24)		–7 (–18 to 4)	
	Women	137/524 (26.1)		71,053/335,548 (21.2)		–4.9 (–13 to 3.1)	
**Race**							
	Black or African American	54/152 (35.7)		45,513/148,120 (30.7)		–5 (–11.1 to 1.2)	
	White	187/702 (26.6)		67,594/308,224 (21.9)		–4.7 (–7.4 to –2)^f^	
	Other	18/80 (22.6)		8,944/91,954 (9.7)		–12.9 (–19.5 to –6.2)	
**Age group**							
	18-39	21/197 (10.8)		34,282/214,685 (16)		5.2 (2.1 to 8.2)^f^	
	40-64	133/406 (32.8)		60,657/240,084 (25.3)		–7.5 (–11.2 to –3.8)	
	65+	204/331 (61.6)		25,699/88,569 (29)		–32.6 (–36.3 to –28.9)	
**Men by race**							
	Black or African American	24/60 (40.6)		17,678/56,004 (31.6)		–9 (–15.2 to –2.9)	
	White	91/314 (29.1)		29,448/120,672 (24.4)		–4.7 (–8.8 to –0.6)^f^	
	Other	9/36 (24.1)		3,871/36,008 (10.8)		–13.3 (–23.2 to –3.5)	
**Women by race**							
	Black or African American	30/92 (32.2)		27,835/92,113 (20.3)		–4.1 (–7.4 to –0.7)^f^	
	White	95/388 (24.4)		38,146/187,541 (9.1)		–11.7 (–20.4 to –3)	
	Other	9/44 (20.8)		5,072/55,894 (30.2)		–2 (–9.9 to 6)^f^	
**Men by age group**							
	18-39	18/99 (18.5)		13,875/77,992 (17.8)		–0.7 (–6.2 to 4.8)^f^	
	40-64	56/178 (31.2)		27,100/98,778 (27.4)		–3.8 (–9.1 to 1.6)^f^	
	65+	90/133 (67.4)		9,694/34,606 (28)		–39.4 (–45.1 to –33.6)	
**Women by age group**							
	18-39	3/98 (3.4)		20,407/136,663 (14.9)		11.5 (9.2 to 13.8)	
	40-64	78/228 (34.2)		33,556/141,286 (23.8)		–10.4 (–15.7 to –5.2)	
	65+	114/198 (57.5)		16,005/53,954 (29.7)		–27.8 (–35.1 to –20.5)	

^a^BRFSS: Behavioral Risk Factor Surveillance System.

^b^Sample size=934.

^c^Indiana Network for Patient Care.

^d^Sample size=548,298.

^e^Δ: mean difference.

^f^Behavioral Risk Factor Surveillance System and Indiana Network for Patient Care phenotypes were determined as statistically equivalent by the two 1-sided *t* test method.

**Table 4:** Phenotype 5, overall ≥1 clinical diagnosis or ≥1 vitals indicated. This table depicts the full analytical results for P5 at the 80% CI.

Characteristic		BRFSS^a,b^, n/N (%)	INPC^c,d^, n/N (%)	%Δ^e^ (Δ80% CI)
Overall		235/934 (28.4)	151,645/548,298 (27.7)	–0.7 (–3.2 to 1.7)^f^
**Gender**				
	Men	127/410 (31)	63,992/212,684 (30.1)	–0.9 (–11.9 to 10.1)
	Women	137/524 (26.1)	87,652/335,548 (26.1)	0 (–8 to 8.1)^f^
**Race**				
	Black or African American	54/152 (35.7)	71,464/148,120 (48.2)	12.5 (6.4 to 18.7)
	White	187/702 (26.6)	137,674/308,224 (44.7)	18.1 (15.4 to 20.8)
	Other	18/80 (22.6)	31,158/91,954 (33.9)	11.3 (4.6 to 17.9)
**Age group (y)**				
	18-39	21/197 (10.8)	36,157/214,685 (16.8)	6 (3 to 9.1)^f^
	40-64	133/406 (32.8)	74,864/240,084 (31.2)	–1.6 (–5.3 to 2.1)^f^
	65+	204/331 (61.6)	38,356/88,569 (43.3)	–18.3 (–22 to –14.6)
**Men by race**				
	Black or African American	24/60 (40.6)	21,091/56,004 (37.7)	–2.9 (–9.1 to 3.2)^f^
	White	91/314 (29.1)	37,622/120,672 (31.2)	2.1 (–2 to 6.2)^f^
	Other	9/36 (24.1)	5,268/36,008 (14.6)	–9.5 (–19.3 to 0.4)
**Women by race**				
	Black or African American	30/92 (32.2)	30,285/88,868 (34.1)	1.9 (–5.1 to 1.6)^f^
	White	95/388 (24.4)	41,094/181,412 (22.7)	–1.7 (–6.1 to 9.8)^f^
	Other	9/44 (20.8)	5,959/54,954 (10.8)	–10 (–18.7 to –1.3)
**Men by age group (y)**				
	18-39	18/99 (18.5)	14,819/77,992 (19)	0.5 (–5 to 6)^f^
	40-64	56/178 (31.2)	33,567/98,778 (34)	2.8 (–2.6 to 8.2)^f^
	65+	90/133 (67.4)	15,011/34,606 (43.4)	–24 (–29.8 to –18.3)
**Women by age group (y)**				
	18-39	3/98 (3.4)	21,331/136,663 (15.6)	12.2 (9.9 to 14.5)
	40-64	78/228 (34.2)	41,296/141,286 (29.2)	–5 (–10.2 to 0.3)
	65+	114/198 (57.5)	23,345/53,954 (43.3)	–14.2 (–21.5 to –6.9)

^a^BRFSS: Behavioral Risk Factor Surveillance System.

^b^Sample size=934.

^c^Indiana Network for Patient Care.

^d^Sample size=548,298.

^e^Δ: mean difference.

^f^Behavioral Risk Factor Surveillance System and Indiana Network for Patient Care phenotypes were determined as statistically equivalent by the two 1-sided *t* test method.

## Discussion

### Principal Findings

Our study examined the prevalence estimates of 6 distinct EHR-based phenotypes to ascertain whether EHR-derived estimates are equivalent to estimates produced by survey methods. The 2 clinical phenotypes (P2 and P5) relying primarily on vital statistics data showed the closest equivalence to BRFSS hypertension prevalence estimates. This suggests that clinical variables, such as blood pressure readings, are important in classifying hypertension cases when compared to national survey data. However, clinical measurements are often missing from national surveys (eg, BRFSS). When clinical measurements are present (eg, the National Health and Nutrition Examination Survey), the survey possesses an even smaller sample size and is frequently more costly. Establishing robust local prevalence estimates may require local health departments to capture blood pressure measurements, which is cost prohibitive. EHR data may provide a more economical approach to the collection of clinical measurements. Additionally, EHRs can supply these measurements regularly forgoing the need for additional, specific public health data collection efforts.

Interestingly, phenotypes that relied on diagnosis code data performed less robustly. Previous studies have demonstrated the underreporting of conditions when relying on diagnostic codes alone [22-24]. Accordingly, it is possible that diagnostic codes themselves are not sensitive enough for identification of hypertension. Further, 1 possible reason for this is the type of encounter for which an individual is seen. For example, if the patient is being seen primarily in emergency or inpatient settings, a diagnosis of hypertension may not be coded, but the vital measurements would be available.

In our results, P6, which is the broadest and most sensitive definition of hypertension [21], did not align with the BRFSS at the overall population level. The hypertension BRFSS instrument item asks “has a doctor told you that you have hypertension?” [20]. This allows for variability in interpretation and may include individuals with a single elevated blood pressure incident or someone who is prehypertensive. Accordingly, it is logical that a computable phenotype using a combination of clinical data elements would be more sensitive to a diagnosis of hypertension but not to the broad question posed by the BRFSS. However, the phenotypes using a variety of clinical measurements may be a more robust measurement of hypertension for local health departments to deploy.

The results showcase the importance of the inclusion of vital statistics, which proved more sensitive for overall comparison and certain subpopulations when the CI threshold was lower. The results of P6 being associated with lower CIs were not surprising given the smaller sample sizes inherent in analyses of subpopulations. Compared to estimates from survey data, more numerous records available in the HIE or multiple EHR systems would allow for smaller CIs in estimates about subpopulations.

While not all algorithms demonstrated equivalency, 2 of the phenotypes demonstrated the potential for EHR data to provide prevalence estimates that are likely to be within 10 percentage points of BRFSS estimates. Accordingly, the use of EHR data may be a better option to estimate disease burden than costly community health surveys. EHR data have several benefits. First, EHR-derived prevalence estimates are timelier. This methodology can be implemented regularly (eg, quarterly and semiannually) to address the needs of the community compared to national surveys. National surveys are typically conducted annually and require time for postprocessing for data. These conditions result in delayed estimates, making the data untimely for certain population health questions. For certain conditions and interventions, this may prove useful for the identification of community needs as well as the timely assessment of community-level interventions. For example, we are using these methods to estimate changes in childhood obesity in multiple urban neighborhoods that received community-level interventions to address childhood obesity [25].

Second, the EHR-derived measures can be tailored to the specific needs of local health departments. Working in coordination with health care systems or HIE networks, local health departments may arrange to receive the data most relevant to their specific question rather than using proxy constructs from national data. Additionally, the EHR-based measures were manually validated and demonstrated to be of high quality, showing strong specificity and positive predictive values [21]. As reported in the results, the computable phenotypes identified a higher prevalence for the Black or African American community. Some of this variation could be attributed to the overrepresentation of inner-city health system patients within the County. However, the demographic analysis supports the premise that the BRFSS may be underrepresentative of the Black or African American population. This argument may be bolstered by the higher prevalence of subpopulations represented within the INPC demographics, both the overall cohort and the hypertension cohort. High-quality estimates, partnered with customization to local needs, will ultimately provide more robust measures for the local health departments.

Further, 1 limitation in the broader use of this methodology is most public health agencies’ lack of legal authority to require reporting of data about chronic conditions. Currently, hospitals are not required to report clinical measurements or metrics related to chronic diseases, such as hypertension, to public health authorities beyond discharge data. Discharge data primarily consist of diagnostic codes, which may not reliably capture chronic disease burden as discussed above. Currently, the reporting of these data is voluntary and, therefore, unlikely to occur given the resources, human, and technological requirements to do so on the part of providers. However, HIE networks (such as INPC) have existing infrastructures that can be leveraged to address community surveillance needs. Data are already aggregated across health care systems and providers within the community, addressing a large amount of the work required to implement surveillance of chronic conditions. This analysis suggests support for leveraging HIE networks in the community for chronic disease surveillance.

The widening use of the Fast Healthcare Interoperability Resources standard and the Trusted Exchange Framework and Common Agreement for health data exchange may also increase public health agencies’ opportunity to access EHR data [26,27]. There are still barriers to the full adoption of HIE networks into the public health environment, such as infrastructure [28] and data quality [29]. However, the COVID-19 pandemic revealed the role HIE could play in support of public health needs [17]. This is increasingly becoming important given the burden of post–COVID-19 conditions [30] and the potential increase in chronic conditions after the pandemic. Surveillance of chronic conditions is critical to public health practice. The efforts to modernize the nation’s public health infrastructure, which are currently underway, should consider the important role HIE networks can play in support of chronic disease surveillance. Admittedly, future work will involve the implementation of HIE networks in those areas of the United States where they are not currently present.

A second limitation is the inconsistent and imprecise equivalency we have demonstrated between the HIE and BRFSS estimates. The BRFSS estimates themselves are fairly imprecise even for a population of about 1 million, as in Marion County, and so make a weak “gold standard,” especially for subpopulations. Conversely, EHR data only reflect persons with health care encounters, and persons with frequent visits are more likely to have enough EHR data to satisfy some phenotype definition. With health care use varying by health status, race, age, employment, and other factors, EHR data would need adjustment for systematic biases before being interpreted as representative of the general community or subpopulations of interest. Further research would reveal what adjustments can improve how well EHR-based estimates approximate population health statistics. This study is subject to limitations related to the quantity and type of available data. Equivalence may be improved by a more complete capture of an area’s health care providers, especially in ambulatory and primary care settings. Improved data capture would increase the EHR-based prevalence estimates. Data might be weighted according to patient characteristics, such as race, age, gender, or type of health insurance, allowing estimates to be adjusted to be more representative of the general population.

As noted above, this study is subject to limitations related to data availability, namely the period for which comprehensive data was available. There have been advancements in EHR adoption and use in the period from 2014 to now. EHR and HIE adoption will continue to be advanced by data modernization activities, which have in turn been spurred by gaps identified in the COVID-19 pandemic. The data availability of important measurements such as vitals, medications, and diagnoses will likely become routinely captured and shared as part of these activities. This suggests, and more recent literature suggests, that the accuracy of computable phenotypes may improve with these advancements [31,32].

### Conclusions

This study demonstrates the feasibility of using EHR-derived prevalence estimates as rough substitutes for population-based survey estimates at the community level. It highlights the importance of critically assessing which data elements to include when deriving the EHR-based estimates. Using comprehensive data sources, containing complete clinical data as well as data representative of the population, may enhance local estimates. The number of people represented in EHR data versus survey data may allow for locally accurate EHR-based measurements of subpopulations. This is critical when considering health disparities as more robust measurements for subpopulations may enable targeted public health interventions.

## Appendix

Multimedia Appendix 1 Two 1-sided *t* test analyses at 80% CI.

Multimedia Appendix 2 Two 1-sided *t* test analyses at 90% CI.

## Abbreviations

BRFSS: Behavioral Risk Factor Surveillance System
EHR: electronic health record
HIE: health information exchange
INPC: Indiana Network for Patient Care
NYC: New York City
TOST: two 1-sided *t* test

## Data Availability

The data sets generated or analyzed during this study are not publicly available due to privacy and governance concerns but are available from the corresponding author upon reasonable request and with the completion of appropriate governance.

## References

[1] Key facts about hypertension. World Health Organization.

[2] Merai R, Siegel C, Rakotz M, Basch P, Wright J, Wong B, DHSc, Phoebe Thorpe (2016). CDC grand rounds: a public health approach to detect and control hypertension. MMWR Morb Mortal Wkly Rep.

[3] MacLeod KE, Ye Z, Donald B, Wang G (2022). A literature review of productivity loss associated with hypertension in the United States. Popul Health Manag.

[4] Singh JA, Yu S (2016). Emergency department and inpatient healthcare utilization due to hypertension. BMC Health Serv Res.

[5] National Center for Health Statistics (2022). National center for health statistics mortality data on CDC WONDER. Centers for Disease Control and Prevention.

[6] Mozaffarian D, Benjamin EJ, Go AS, Arnett DK, Blaha MJ, Cushman M, de Ferranti S, Després JP, Fullerton HJ, Howard VJ, Huffman MD, Judd SE, Kissela BM, Lackland DT, Lichtman JH, Lisabeth LD, Liu S, Mackey RH, Matchar DB, McGuire DK, Mohler ER, Moy CS, Muntner P, Mussolino ME, Nasir K, Neumar RW, Nichol G, Palaniappan L, Pandey DK, Reeves MJ, Rodriguez CJ, Sorlie PD, Stein J, Towfighi A, Turan TN, Virani SS, Willey JZ, Woo D, Yeh RW, Turner MB (2015). Heart disease and stroke statistics—2015 update: a report from the American Heart Association. Circulation.

[7] Ebinger JE, Driver M, Joung S, Tran T, Barajas D, Wu M, Botting PG, Navarrette J, Sun N, Cheng S (2022). Hypertension and excess risk for severe COVID-19 illness despite booster vaccination. Hypertension.

[8] Chakraborty DS, Lahiry S, Choudhury S (2021). Hypertension clinical practice guidelines (ISH, 2020): what is new?. Med Princ Pract.

[9] Jamoom EW, Yang N, Hing E (2016). Adoption of certified electronic health record systems and electronic information sharing in physician offices: United States, 2013 and 2014. NCHS Data Brief.

[10] Smith S, Saunders R, Stuckhardt L, McGinnis JM, Institute of Medicine (U.S.), Committee on the Learning Health Care System in America (2013). Best Care at Lower Cost: The Path to Continuously Learning Health Care in America.

[11] Callado M, Desai R, Dugan M, Jorling E Health data for action. AcademyHealth.

[12] Chan KS, Fowles JB, Weiner JP (2010). Review: electronic health records and the reliability and validity of quality measures: a review of the literature. Med Care Res Rev.

[13] Newton-Dame R, McVeigh KH, Schreibstein L, Perlman S, Lurie-Moroni E, Jacobson L, Greene C, Snell E, Thorpe LE (2016). Design of the New York City Macroscope: innovations in population health surveillance using electronic health records. EGEMS (Wash DC).

[14] Perlman SE, McVeigh KH, Thorpe LE, Jacobson L, Greene CM, Gwynn RC (2017). Innovations in population health surveillance: using electronic health records for chronic disease surveillance. Am J Public Health.

[15] Community Health Survey. NYC Health.

[16] Madhavan S, Bastarache L, Brown JS, Butte AJ, Dorr DA, Embi PJ, Friedman CP, Johnson KB, Moore JH, Kohane IS, Payne PRO, Tenenbaum JD, Weiner MG, Wilcox AB, Ohno-Machado L (2021). Use of electronic health records to support a public health response to the COVID-19 pandemic in the United States: a perspective from 15 academic medical centers. J Am Med Inform Assoc.

[17] Dixon BE, Grannis SJ, McAndrews C, Broyles AA, Mikels-Carrasco W, Wiensch A, Williams JL, Tachinardi U, Embi PJ (2021). Leveraging data visualization and a statewide health information exchange to support COVID-19 surveillance and response: application of public health informatics. J Am Med Inform Assoc.

[18] Dixon BE, Tao G, Wang J, Tu W, Hoover S, Zhang Z, Batteiger TA, Arno JN (2017). An integrated surveillance system to examine testing, services, and outcomes for sexually transmitted diseases. Stud Health Technol Inform.

[19] (2015). Behavioral Risk Factor Surveillance System: weighting BRFSS data. Centers for Disease Control and Prevention.

[20] Behavioral Risk Factor Surveillance System. Centers for Disease Control and Prevention.

[21] Valvi N, McFarlane T, Allen KS, Gibson PJ, Dixon B (2023). Identification of hypertension in electronic health records through computable phenotype development and validation for use in public health surveillance: retrospective study. JMIR Form Res.

[22] Khera R, Mortazavi BJ, Sangha V, Warner F, Young HP, Ross JS, Shah ND, Theel ES, Jenkinson WG, Knepper C, Wang K, Peaper D, Martinello RA, Brandt CA, Lin Z, Ko AI, Krumholz HM, Pollock BD, Schulz WL (2022). A multicenter evaluation of computable phenotyping approaches for SARS-CoV-2 infection and COVID-19 hospitalizations. NPJ Digit Med.

[23] Pfaff ER, Girvin AT, Bennett TD, Bhatia A, Brooks IM, Deer RR, Dekermanjian JP, Jolley SE, Kahn MG, Kostka K, McMurry JA, Moffitt R, Walden A, Chute CG, Haendel MA (2022). Identifying who has long COVID in the USA: a machine learning approach using N3C data. Lancet Digit Health.

[24] Festa N, Shi SM, Kim DH (2020). Accuracy of diagnosis and health service codes in identifying frailty in Medicare data. BMC Geriatr.

[25] Duszynski T, Crago J, Hancock A, Valvi N, Luo A (2023). Weight trends among children and adolescents within Central Indiana. Jump IN.

[26] Miliard M National Coordinator: TEFCA will enable 'North Star' architecture for public health. HIMSS22 Healthcare IT News.

[27] Trusted Exchange Framework and Common Agreement (TEFCA). HealthIT.gov.

[28] Khan S, Shea CM, Qudsi HK (2017). Barriers to local public health chronic disease surveillance through health information exchange: a capacity assessment of health departments in the health information network of South Texas. J Public Health Manag Pract.

[29] Horth RZ, Wagstaff S, Jeppson T, Patel V, McClellan J, Bissonette N, Friedrichs M, Dunn AC (2019). Use of electronic health records from a statewide health information exchange to support public health surveillance of diabetes and hypertension. BMC Public Health.

[30] Han Q, Zheng B, Daines L, Sheikh A (2022). Long-term sequelae of COVID-19: a systematic review and meta-analysis of one-year follow-up studies on post-COVID symptoms. Pathogens.

[31] Hohman KH, Zambarano B, Klompas M, Wall HK, Kraus EM, Carton TW, Jackson SL (2023). Development of a hypertension electronic phenotype for chronic disease surveillance in electronic health records: key analytic decisions and their effects. Prev Chronic Dis.

[32] Kraus EM, Brand B, Hohman KH, Baker EL (2022). New directions in public health surveillance: using electronic health records to monitor chronic disease. J Public Health Manag Pract.

