# Supplementary material for: Electronic Health Records for Population Health Management: Comparison of Electronic Health Record–Derived Hypertension Prevalence Measures Against Established Survey Data
Source: Online J Public Health Inform. 2024 Mar 13;16:e48300. doi: 10.2196/48300 (PMC10973965; doi:10.2196/48300)
Supplement: Multimedia Appendix 2 [file ojphi_v16i1e48300_app2.docx]

**Multimedia Appendix 2.** Two 1-sided *t* test analyses at 90% confidence interval.

For all presented tables, the following footnotes apply:

^a^Sample size = 934

^b^Sample size = 548,298

^c^BRFSS & INPC phenotypes determined statistically equivalent by TOST method

^d^AA=African American;

^e^ Δ=Mean difference

**Table S1.** Phenotype 1 ≥1 clinical diagnosis^b^.

|  | **BRFSS %** | **%** | **%Δ^c^** | **Δ80% CI** |
| --- | --- | --- | --- | --- |
| **Overall** | 28.4 | 16.5 | –11.9 | (–15-–8.8) |
| **Sex** | | | | |
| Male | 31 | 18 | –13 | (–27.1-1.1) |
| Female | 26.1 | 15.6 | –10.5 | (–20.8-–0.2) |
| **Race** | | | | |
| Black/African American | 35.7 | 35.7 | 22.6 | –13.1 |
| White | 26.6 | 26.6 | 16.3 | –10.3 |
| Other | 22.6 | 22.6 | 7.8 | –14.8 |
| **Age** | | | | |
| 18-39 | 10.8 | 10.8 | 3.8 | –7 |
| 40-64 | 32.8 | 32.8 | 20.2 | –12.6 |
| 65+ | 61.6 | 61.6 | 36.3 | –25.3 |
| **Male by Race** | | | | |
| Black/African American | 40.6 | 22.3 | –18.3 | (–26.2-–10.5) |
| White | 29.1 | 18.9 | –10.2 | (–15.5-–5) |
| Other | 24.1 | 8.5 | –15.6 | (–28.2-–3) |
| **Female by Race** | | | | |
| Race | 32.2 | 22.7 | –9.5 | (–19.6-0.7) |
| Black/African American | 24.4 | 14.6 | –9.8 | (–14.1-–5.6) |
| White | 20.8 | 7.3 | –13.5 | (–24.7-–2.4) |
| **Male by Age** | | | | |
| 18-39 | 18.5 | 18.5 | –13.8 | (–20.9-–6.8) |
| 40-64 | 31.2 | 31.2 | –9.3 | (–16.2-–2.4) |
| 65+ | 67.4 | 67.4 | –31.2 | (–38.6-–23.8) |
| **Female by Age** | | | | |
| 18-39 | 3.4 | 3.3 | –0.1 | (–3.1-2.8)^c^ |
| 40-64 | 34.2 | 19 | –15.2 | (–21.9-–8.5) |
| 65+ | 57.5 | 36.4 | –21.1 | (–30.5-–11.8) |

**Table S2.** Phenotype 2 ≥1 vitals indicated overall^b^.

|  | **BRFSS %** | **%** | **%Δ^c^** | **Δ80% CI** |
| --- | --- | --- | --- | --- |
| **Overall** | 28.4 | 29.1 | 0.7 | (–2.5-3.8)^c^ |
| **Sex** | | | | |
| Male | 31 | 31.4 | 0.4 | (–13.7-14.5) |
| Female | 26.1 | 27.6 | 1.5 | (–8.8-11.8) |
| **Race** | | | | |
| Black/African American | 35.7 | 38.5 | 2.8 | (–5.1-10.7) |
| White | 26.6 | 28.9 | 2.3 | (–1.1-5.8) |
| Other | 22.6 | 14.2 | –8.4 | (–16.9-0.2) |
| **Age** | | | | |
| 18-39 | 10.8 | 23.1 | 12.3 | (8.4-16.3) |
| 40-64 | 32.8 | 32 | –0.8 | (–5.6-3.9) |
| 65+ | 61.6 | 35.3 | –26.3 | (–31.1-–21.6) |
| **Male by Race** | | | | |
| Black/African American | 40.6 | 39.7 | –0.9 | (–8.8-7) |
| White | 29.1 | 32.2 | 3.1 | (–2.2-8.3) |
| Other | 24.1 | 15.8 | –8.3 | (–20.9-4.4) |
| **Female by Race** | | | | |
| Race | 32.2 | 37.8 | 5.6 | (–4.6-15.7) |
| Black/African American | 24.4 | 26.9 | 2.5 | (–1.8-6.7) |
| White | 20.8 | 13.2 | –7.6 | (–18.7-3.6) |
| **Male by Age** | | | | |
| 18-39 | 18.5 | 26.3 | 7.8 | (0.7-14.8) |
| 40-64 | 31.2 | 34.3 | 3.1 | (–3.7-10) |
| 65+ | 67.4 | 34.6 | –32.8 | (–40.2-–25.5) |
| **Female by Age** | | | | |
| 18-39 | 3.4 | 21.3 | 11.5 | (15-20.9) |
| 40-64 | 34.2 | 30.3 | –3.9 | (–10.6-2.9) |
| 65+ | 57.5 | 35.7 | –21.8 | (–31.1-–12.4) |

**Table S3.** Phenotype 3 ≥2 vitals indicated^b^.

|  | **BRFSS %** | **%** | **%Δ^c^** | **Δ80% CI** |
| --- | --- | --- | --- | --- |
| **Overall** | 28.4 | 22.3 | –6.1 | (–9.3-–3) |
| **Sex** | | | | |
| Male | 31 | 24 | –7 | (–21.1-7.1) |
| Female | 26.1 | 21.2 | –4.9 | (–15.3-5.4) |
| **Race** | | | | |
| Black/African American | 35.7 | 30.7 | –5 | (–12.8-2.9) |
| White | 26.6 | 21.9 | –4.7 | (–8.1-–1.2) |
| Other | 22.6 | 9.7 | –12.9 | (–21.4-–4.3) |
| **Age** | | | | |
| 18-39 | 10.8 | 16 | 5.2 | (1.2-9.1) |
| 40-64 | 32.8 | 25.3 | –7.5 | (–12.3-–2.8) |
| 65+ | 61.6 | 29 | –32.6 | (–37.3-–27.8) |
| **Male by Race** | | | | |
| Black/African American | 40.6 | 31.6 | –9 | (–16.9-–1.2) |
| White | 29.1 | 24.4 | –4.7 | (–9.9-0.6) |
| Other | 24.1 | 10.8 | –13.3 | (–26-–0.7) |
| **Female by Race** | | | | |
| Race | 32.2 | 30.2 | –2 | (–12.1-8.2) |
| Black/African American | 24.4 | 20.3 | –4.1 | (–8.3-0.2) |
| White | 20.8 | 9.1 | –11.7 | (–22.9-–0.6) |
| **Male by Age** | | | | |
| 18-39 | 18.5 | 17.8 | –0.7 | (–7.8-6.3) |
| 40-64 | 31.2 | 27.4 | –3.8 | (–10.7-3.1) |
| 65+ | 67.4 | 28 | –39.4 | (–46.8-–32) |
| **Female by Age** | | | | |
| 18-39 | 3.4 | 0.4 | 11.5 | (8.6-14.5) |
| 40-64 | 34.2 | 0.4 | –10.4 | (–17.2-–3.7) |
| 65+ | 57.5 | 0.5 | –27.8 | (–37.2-–18.5) |

**Table S4.** Phenotype 4 ≥1 clinical diagnosis and ≥1 vitals indicated^b^.

|  | **BRFSS %** | **%** | **%Δ^c^** | **Δ80% CI** |
| --- | --- | --- | --- | --- |
| **Overall** | 28.4 | 11.6 | –16.8 | (–19.9-–13.6) |
| **Sex** | | | | |
| Male | 31 | 12.5 | –18.5 | (–32.6-–4.4) |
| Female | 26.1 | 11.1 | –15 | (–25.3-–4.7) |
| **Race** | | | | |
| Black/African American | 35.7 | 17.4 | –18.3 | (–26.2-–10.4) |
| White | 26.6 | 11 | –15.6 | (–19-–12.1) |
| Other | 22.6 | 4.4 | –18.2 | (–26.7-–9.7) |
| **Age** | | | | |
| 18-39 | 10.8 | 2.9 | –7.9 | (–11.8-–3.9) |
| 40-64 | 32.8 | 14.9 | –17.9 | (–22.7-–13.2) |
| 65+ | 61.6 | 23.5 | –38.1 | (–42.9-–33.4) |
| **Male by Race** | | | | |
| Black/African American | 40.6 | 17.1 | –23.5 | (–31.3-–15.6) |
| White | 29.1 | 12.6 | –16.5 | (–21.8-–11.3) |
| Other | 24.1 | 4.8 | –19.3 | (–32-–6.7) |
| **Female by Race** | | | | |
| Race | 32.2 | 17.5 | –14.7 | (–24.8-–4.5) |
| Black/African American | 24.4 | 10 | –14.4 | (–18.6-–10.1) |
| White | 20.8 | 4.2 | –16.6 | (–27.8-–5.5) |
| **Male by Age** | | | | |
| 18-39 | 18.5 | 3.5 | –15 | (–22-–7.9) |
| 40-64 | 31.2 | 16 | –15.2 | (–22.1-–8.3) |
| 65+ | 67.4 | 22.3 | –45.1 | (–52.5-–37.7) |
| **Female by Age** | | | | |
| 18-39 | 3.4 | 2.6 | –0.8 | (–3.8-2.1)^c^ |
| 40-64 | 34.2 | 14.1 | –20.1 | (–26.9-–13.4) |
| 65+ | 57.5 | 24.2 | –33.3 | (–42.7-–24) |

**Table S5.** Phenotype 5 ≥1 clinical diagnosis or ≥1 vitals indicated^b^.

|  | **BRFSS %** | **%** | **%Δ^c^** | **Δ80% CI** |
| --- | --- | --- | --- | --- |
| **Overall** | 27.7 | –0.7 | (–3.9-2.4)^c^ | 27.7 |
| **Sex** | | | | |
| Male | 31 | 30.1 | –0.9 | (–15-13.2) |
| Female | 26.1 | 26.1 | 0 | (–10.3-10.4) |
| **Race** | | | | |
| Black/African American | 35.7 | 48.2 | 12.5 | (4.7-20.4) |
| White | 26.6 | 44.7 | 18.1 | (14.6-21.5) |
| Other | 22.6 | 33.9 | 11.3 | (2.8-19.8) |
| **Age** | | | | |
| 18-39 | 10.8 | 16.8 | 6 | (2.1-10) |
| 40-64 | 32.8 | 31.2 | –1.6 | (–6.4-3.1) |
| 65+ | 61.6 | 43.3 | –18.3 | (–23-–13.5) |
| **Male by Race** | | | | |
| Black/African American | 40.6 | 37.7 | –2.9 | (–10.8-4.9) |
| White | 29.1 | 31.2 | 2.1 | (–3.2-7.3) |
| Other | 24.1 | 14.6 | –9.5 | (–22.1-3.2) |
| **Female by Race** | | | | |
| Race | 32.2 | 34.1 | 1.9 | (–8.3-12) |
| Black/African American | 24.4 | 22.7 | –1.7 | (–6-2.5) |
| White | 20.8 | 10.8 | –10 | (–21.1-1.2) |
| **Male by Age** | | | | |
| 18-39 | 18.5 | 19 | 0.5 | (–6.6-7.6) |
| 40-64 | 31.2 | 34 | 2.8 | (–4.1-9.7) |
| 65+ | 67.4 | 43.4 | –24 | (–31.4-–16.6) |
| **Female by Age** | | | | |
| 18-39 | 3.4 | 15.6 | 12.2 | (9.3-15.2) |
| 40-64 | 34.2 | 29.2 | –5 | (–11.7-1.8) |
| 65+ | 57.5 | 43.3 | –14.2 | (–23.6-–4.9) |

**Table S6.** Phenotype 6 ≥1 clinical diagnosis or ≥1 vitals indicated or ≥1 medications indicated^b^.

|  | **BRFSS %** | **%** | **%Δ^c^** | **Δ80% CI** |
| --- | --- | --- | --- | --- |
| **Overall** | 27.7 | 38.3 | 9.9 | (7.5-12.4) |
| **Sex** | | | | |
| Male | 31 | 42 | 11 | (0-22) |
| Female | 26.1 | 36 | 9.9 | (1.9-18) |
| **Race** | | | | |
| Black/African American | 35.7 | 47.2 | 11.5 | (5.3-17.6) |
| White | 26.6 | 39.1 | 12.5 | (9.8-15.2) |
| Other | 22.6 | 21.6 | –1 | (–7.7-5.6)^d^ |
| **Age** | | | | |
| 18-39 | 10.8 | 24.6 | 13.8 | (10.7-16.8) |
| 40-64 | 32.8 | 42.1 | 9.3 | (5.6-13) |
| 65+ | 61.6 | 60.4 | –1.2 | (–4.9-2.5)^d^ |
| **Male by Race** | | | | |
| Black/African American | 40.6 | 48.5 | 7.9 | (1.8-14) |
| White | 29.1 | 44.2 | 15.1 | (11-19.2) |
| Other | 24.1 | 24.3 | 0.2 | (–9.7-10) |
| **Female by Race** | | | | |
| Race | 32.2 | 46.4 | 14.2 | (6.3-22.1) |
| Black/African American | 24.4 | 35.8 | 11.4 | (8.1-14.7) |
| White | 20.8 | 19.8 | –1 | (–9.7-7.7)^d^ |
| **Male by Age** | | | | |
| 18-39 | 18.5 | 28.3 | 9.8 | (4.3-15.3) |
| 40-64 | 31.2 | 45.7 | 14.5 | (9.2-19.9) |
| 65+ | 67.4 | 61.4 | –6 | (–11.7-–0.2) |
| **Female by Age** | | | | |
| 18-39 | 3.4 | 22.4 | 19 | (16.7-21.4) |
| 40-64 | 34.2 | 39.6 | 5.4 | (0.1-10.6) |
| 65+ | 57.5 | 59.8 | 2.3 | (–5-9.6)^d^ |
